# Antimicrobial resistance in hospitalized surgical patients: a silently emerging public health concern in Benin

**DOI:** 10.1186/s12941-020-00398-4

**Published:** 2020-11-25

**Authors:** Carine Laurence Yehouenou, Arsène A. Kpangon, Dissou Affolabi, Hector Rodriguez-Villalobos, Françoise Van Bambeke, Olivia Dalleur, Anne Simon

**Affiliations:** 1 Clinical Pharmacy Research Group (CLIP), Louvain Drug Research Institute (LDRI), Université Catholique de Louvain UCLouvain, Brussels, Belgium; 2 Laboratoire de Référence des Mycobactéries (LRM), Cotonou, Benin; 3 Centre National Hospitalier et Universitaire Hubert Koutoukou Maga (CNHU-HKM), Cotonou, Benin; 4 Faculté des Sciences de La Santé (FSS), Université D’Abomey Calavi (UAC), 01BP188, Cotonou, Benin; 5 Ecole Nationale des Techniciens Supérieurs en Santé Publique et Surveillance Epidémiologique, Université de Parakou, Parakou, Benin; 6 Microbiologie, Cliniques Universitaires Saint Luc, Université Catholique de Louvain, UCLouvain, Brussels, Belgium; 7 Pôle de Microbiologie, Institut de Recherche Expérimentale et Clinique (IREC), Université Catholique de Louvain UCLouvain, Brussels, Belgium; 8 Pharmacologie Cellulaire et Moléculaire, Louvain Drug Research Institute (LDRI), Université Catholique de Louvain UCLouvain, Brussels, Belgium; 9 Pharmacy, Clinique Universitaire Saint-Luc, Université Catholique de Louvain, UCLouvain, Brussels, Belgium

**Keywords:** ESBL, Methicillin-resistant *staphylococcus aureus*, Surgical site infection, Multidrug-resistant organisms

## Abstract

**Background:**

Surgical site infections are related to high morbidity, mortality and healthcare costs. Because the emergence of multidrug-resistant bacteria in hospitals is becoming a worldwide challenge for surgeons who treat healthcare-associated infections, we wished to identify the causative agents involved in these infections and the rate of multidrug-resistant bacteria in six public hospitals in Benin.

**Methods:**

Using standard microbiological procedures, we processed pus specimens collected from obstetrics and gastrointestinal surgery wards. Mass spectrometry (MALDI-TOF) was used for confirmation. For the antibiotic susceptibility test, we first used the Kirby-Bauer disk diffusion method. The secondary test (by microdilution) used the Beckton Dickinson Phoenix automated system (Becton Dickinson Diagnostic, USA).

**Results:**

We included 304 patients, whose median length of stay was 9 days. A total of 259 wound swabs (85.2%) had positive aerobic bacterial growth. In obstetrics, *S. aureus* (28.5%, n = 42) was the most common isolate. In contrast, Gram-negative bacteria (GNB) were predominant in gastrointestinal surgery, the most dominant being *E.coli* (38.4%, n = 31). Overall, 90.8% (n = 208) of aerobic bacteria were multidrug resistant. Two-thirds of *S. aureus* (65.3%, n = 32) were methicillin-resistant *Staphylococcus aureus* (MRSA), three of which carried both MRSA and induced clindamycin resistance (ICR). GNB showed high resistance to ceftazidime, ceftriaxone and cefepime. Extended-spectrum beta-lactamases were presented by 69.4% *of E.coli* (n = 43/62) and 83.3% of *K. pneumoniae* (n = 25/30). Overall, twelve Gram-negative bacteria (5.24%) showed resistance to at least one carbapenem. No isolates showed a wild-type susceptible phenotype.

**Conclusion:**

This study shows the alarming prevalence of multidrug-resistant organisms from surgical site infections in Benin hospitals. To reduce the spread of such bacteria in Benin, periodic surveillance of surgical site infections and strict adherence to good hand-hygiene practice are essential.

## Background

Surgical site infections (SSIs) are infections of an incision, organ or space that occur after surgery [1]. In the United States, SSIs are the third most common hospital-acquired infections, accounting for 38% of all nosocomial infections according to the National Nosocomial Infection Surveillance System of the Centers for Disease Control (CDC’s) [2]. In countries with limited resources, SSIs are the most common in the overall patient population, affecting up to 66% of patients who have had an operation—nine times more than in industrialized countries [3]. SSIs increase the length of post-operative hospital stay, the cost of healthcare, and the rate of hospital readmissions [4].

The common bacterial pathogens isolated from SSIs are *Staphylococcus aureus, coagulase negative Staphylococcus* (CoNS), *Acinetobacter *spp.,* Pseudomonas *spp., *Escherichia coli*, *Klebsiella *spp.,* Proteus *spp.,* Enterobacter *spp.,* Citrobacter *spp., and anaerobes such as *Clostridium *spp., and *Peptostreptococcus *spp. [5, 6]*.*The acquisition of antibiotic resistance mechanisms by these bacterial strains has highlighted challenges for the management of SSIs around the world. These challenges have been made even greater by methicillin-resistant *Staphylococcus aureus* (MRSA), extended spectrum beta-lactamases (ESBL) producing Enterobacterales, and the involvement of polymicrobial flora and fungi [7, 8]. ESBL have been reported most often in *Escherichia coli* and *Klebsiella *spp., but also in other bacterial species such as *Pseudomonas aeruginosa,* and *Enterobacter cloacae* [9]*.*

The battle between bacteria and their susceptibility to drugs remains difficult for researchers and clinicians, and also for drug companies that seek effective drugs. SSIs by resistant drug bacteria are also becoming a serious concern in developing countries such as Benin, due mainly to crowded hospitlal environments, the irrational prescription of antimicrobial agents, and poor infection prevention and control programs [10]. Due to these inadequate SSI-surveillance programs, healthcare centers are unable to obtain proper updates on bacteria that are resistant to antimicrobial drugs [11]. The correct antibiotics are expensive and hard to access [12], and the use of broad-spectrum antibiotics is common.

In Benin, most clinical laboratories are not equipped with testing facilities that can detect multidrug-resistant bacteria [13]. Although the treatment process and infection-control measures depend on appropriate knowledge not only of updated antimicrobial therapy, but particularly of pathogens and their resistance, [14] these laboratories have little data on these pathogens’ patterns of antimicrobial resistance. Identifying a bacteria and determining its susceptibility pattern therefore benefits patients, and also helps clinicians select chemotherapies that avoid the emergence of multidrug resistance organisms in hospital [15]. To generate locally applicable data and guide empirical therapy in areas where culture and drug-susceptibility testing facilities are scarce, this study thus sought to determine the magnitude of multidrug-resistant bacteria identified from SSIs.

## Methods and materials

### Study design and setting

This cross-sectional study was designed and conducted explicitly to determine the bacteriological profile of aerobes isolated from SSIs. These isolates were identified, and antimicrobial susceptibility testing was performed, in order to analyze the prevalence of multidrug-resistant organisms (MDRO) and particular phenotypes: MRSA, ESBL, and carbapenemase producing bacteria (CPO).

Patients who consented to participate in the study were included between January 2019 and January 2020. Patients who had had initial surgery in another hospital or ward (internal medicine or emergency), or had had prior to antibiotic treatment, and those who did not volunteer to participate were excluded from the study. We included the obstretrics wards (particularly ceasarean sections) and gastrointestinal wards at six public hospitals in Benin: *Bethesda, Centre Hospitalier Universitaire de Zone de Suru Lere, Centre National Hospitalier Université Hubert Koutoukou Maga* (CNHU-HKM), *Centre Hospitalier Universitaire de la Mère et L’enfant* (CHUMEL), *Centre Hospitalier Universitaire Départemental Ouéme/Plateau* (CHUDOP) and *Centre Hospitalier Universitaire de Zone d’Abomey Calavi.* Our choice of wards lay in the facts that caesarean sections are one of the most frequent surgical procedures worldwide [16], and that gastrointestinal section is available in these six public hospitals. All participating hospitals were located in the south of Benin, thereby allowing daily transport from each hospital to the CNHU-HKM laboratory, where all wound swabs collected were analyzed. All results were confirmed in the laboratory of the *Cliniques Universitaires Saint-Luc-UCLouvain *(Brussels, Belgium).

### Sampling and collection method

The case definitions and clinical criteria of SSIs (superficial incisional SSI, deep incisional SSI, and organ/space SSI) were taken from the guidelines of the Centers for Disease Control and Prevention on the prevention of SSI [2]. A preliminary step in the project consisted of training nurses in the sampling technique. These nurses then collected all the samples per hospital. On a day when a clinical SSI was detected, one swab was collected aseptically from each infected patient using a sterile cotton swab. Each sample was labeled with the date of sample collection, the collection method, and the patient’s details. The swab was immediately dipped into a sterile tube with transport medium (Amies, Beckton Dickinson) and delivered to the bacteriology laboratory at CNHU-HKM. Coagulase-negative *staphylococcus aureus* were considered as pathogens only when isolated in two consecutive sampling swabs.

Socio-demographics and clinical data were obtained from the patients’ files and by physical examination using a structured questionnaire. The following data were collected: ward to which admitted, age, gender, history of illness, and antibiotics used during and after surgery. Before the actual data was collected, a pretest of the data collection instrument was conducted to ensure the appropriateness of the questionnaire. If necessary, modifications were made. Data collection was supervised daily by the research team.

## Processing of samples

### Macroscopic and microscopic examination of samples

All the specimens were visually examined for consistency, color, turbidity, and the presence or absence of blood. Gram staining of each specimen was performed [17].

### Culture of specimens and isolation of bacteria

Bacterial identification was performed according to the guidelines for microbiological methods of the European Committee of Antimicrobial Susceptibility Testing guidelines [18]. Cultures were incubated for a total of 48 h (if there was no growth at 24 h) at 37 °C in aerobic atmosphere, and then examined for microbial growth. Identification of Gram-positive bacteria was done using Gram staining, and the catalase test, coagulase test, DNase test and Pastorex staphylococci plus test (Pastorex, staph plus Biorad). Gram-negative strains were identified using various biochemical tests: oxidase, and characteristics of the Analytical Profile Index (API 20E, Biomerieux, Lyon) such as the Voges Proskauer (VP) test, indole test and citrate utilization.

All identifications were confirmed in Belgium by using Matrix Assisted Laser Desorption Ionization-Time of Flight (MALDI-TOF) mass spectrometry (Brucker Daltonics, Bremen, Germany). Due to budgetary constraints and a lack of laboratory facilities, we were unable to investigate anaerobic bacteria.

### Antimicrobial susceptibility test (AST)

Antimicrobial susceptibility testing was performed for all isolates according to the modified Kirby Bauer disk diffusion technique as described in the European Committee on Antimicrobial Susceptibility Testing guidelines [18]. Antibiotics were purchased from BioRad (Marnes-la-Coquette, France) and included for *S. aureus:* ampicillin (10 $\upmu \text{g})$, cefotaxime (30 g$\upmu$), cefoxitin (30 ${\upmu {\text{g}}})$, gentamicin (10 ${\upmu {\text{g}}})$,amikacin (30 ${\upmu {\text{g}}}$), ciprofloxacin (5 ${\upmu {\text{g}}}$), trimethoprim + sulfamethoxazole (25 g$\upmu$), tetracycline (30 g$\upmu$), and chloramphenicol (30 g$\upmu$). The lactose fermenters bacteria were tested for ampicillin (10 ${\upmu {\text{g}}})$, piperacillin (100 ${\upmu {\text{g}}}$), cefotaxime (30 ${\upmu {\text{g}}}$), cefoxitin (30 ${\upmu{\text{g}}}$), ceftriaxone (30 g$\upmu$), gentamicin (10 ${\upmu {\text{g}}}$), tobramycin (10 ${\upmu {\text{g}}}$), amikacin (30 g$\upmu$), ciprofloxacin (5 ${\upmu {\text{g}}})$, trimethoprim + sulfamethoxazole (25 g$\upmu$), imipenem (10 ${\upmu {\text{g}}})$, and meropenem (10 ${\upmu {\text{g}}})$. For lactose non-fermenters bacteria, the disks used included ceftriaxone (30 ${\upmu {\text{g}}})$, ceftazidime (30 ${\upmu {\text{g}}})$, gentamicin (10 ${\upmu {\text{g}}}$), ciprofloxacin and meropenem (10 g$\upmu$). After 24 h of incubation at 37 °C, the inhibition zones were measured and the results were analyzed.

### Phenotypic test for multidrug-resistant bacteria, extended-spectrum beta-lactamases, inducible clindamycin resistant (ICR) in S. aureus, and carbapenemases production

Multidrug resistance was defined as resistance to three or more antimicrobial classes [19]. The presence of ESBL was detected using the double-disk synergy test (DDST) between clavulanate and third-generation cephalosporins and/or aztreonam [20]. Methicillin-resistant *S. aureu*s isolates were detected using the cefoxitin disk (30 g$\mu$) method. The diameter of the zone of inhibition for cefoxitin was < 21 mm [21]. Similarly, inducible macrolide-lincosamide streptogramin-B (iMLS_B_) resistance was detected in *S. aureus* with the D-test disk method using clindamycin (2  ug) and erythromycin (15 ug) on MHA plates. After overnight incubation, isolates with a flattened zone of inhibition adjacent to the erythromycin disk (referred to as a “D” zone) were considered to exhibit inducible clindamycin resistance [22]. The presence of resistance to at least one carbapenem was checked with the RESIST-3O.K.N.ICT (Coris Bioconcept, Gembloux, Belgium), which detects OXA 48, KPC and NDM carbapenems. The final results of the ICT test were read when they became positive, at the latest after 15 min. All ESBL, MRSA and CPO strains were confirmed and characterized by whole-genome sequencing.

### Quality control

Standard operating procedures (SOPs) were strictly followed during all bacteriological procedures, starting from sample collection, isolation, identification and antibiotic susceptibility testing. All culture media were prepared according to the manufacturers’ directions, and were checked for their sterility and performance. Two international control bacteria strains, *E. coli ATCC 25922* and S*. aureus ATCC 25923*, were used as reference strains for quality control of the antimicrobial susceptibility and biochemical tests. The same strain of *E.coli* was also considered as a negative control during the screening and phenotypic tests of ESBL-producing lactose fermenters bacteria. In Belgium, the same strain of *E.coli* was also considered as a control for mass spectrometry (MALDI-TOF). For transportation only, we used swabs with transport medium (Amies, Beckton Dickinson).

### Data analysis and statistical tests

Data was entered in Epi-data version 3.1, transferred to Statistical Package for Social Sciences (SPSS) software version 25, and Microsoft Excel software for analysis. Quantitative variables were expressed as median with interquartile range (IQR). A P-value less than 0.05 was considered statistically significant.

#### Results

### Socio-demographic and clinical characteristics

A total of 304 wound swabs were collected from 174 patients with clinical signs of surgical site infections. Obstetrics patients [n = 148; median age 29 years (24-IQR-34)] represented 195 swabs (64.1%). The median length of stay in obstetrics and gastrointestinal surgery was 9 days (6-IQR-14). In gastrointestinal surgery, the patients’ ages ranged from 18 to 76 years, with a median age of 35 years (25-IQR 50). A majority of the patients were female (80.3%). Emergency surgery was the most common type of surgery (82.6%).

### Surgical antimicrobial prophylaxis

Of the samples collected, 172/304 (56.6%) originated from patients who had received preoperative antimicrobial prophylaxis for more than 24 h after surgery. Monotherapy with ceftriaxone was administered to the highest number of patients 57/172 (33%), ampicillin was administered to 23/172 (13%) and amoxicillin/clavulanic acid was administered to 17/172 (9.9%). The most prescribed regimen among the combination regimens was ceftriaxone + metronidazole 35/172 (20%). In both obstetrics and gastrointestinal surgery, there was no difference between the antimicrobial classes used before and after surgery**.**

### Bacterial etiologic agents isolated per ward

**Fig. 1:**
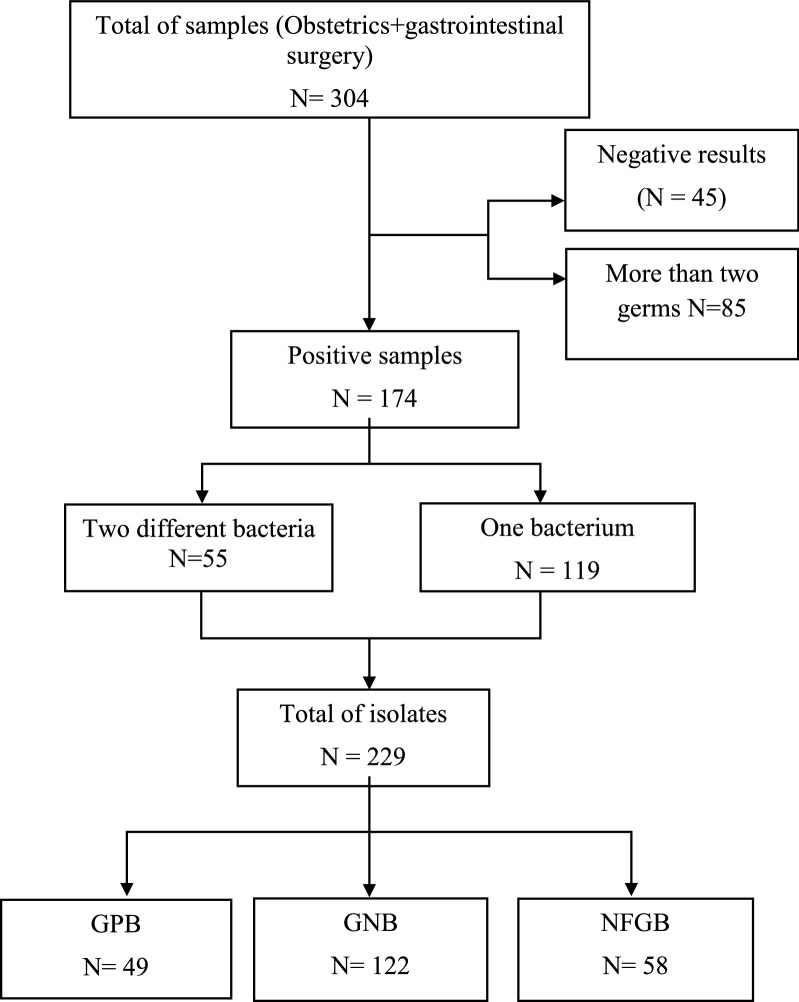
Flow chart showing patient enrollment and data collection**.** GPB: Gram-positive bacteria, GNB: Gram-negative bacteria, NFGB: Non-fermentative Gram-negative bacteria

**Fig. 2:**
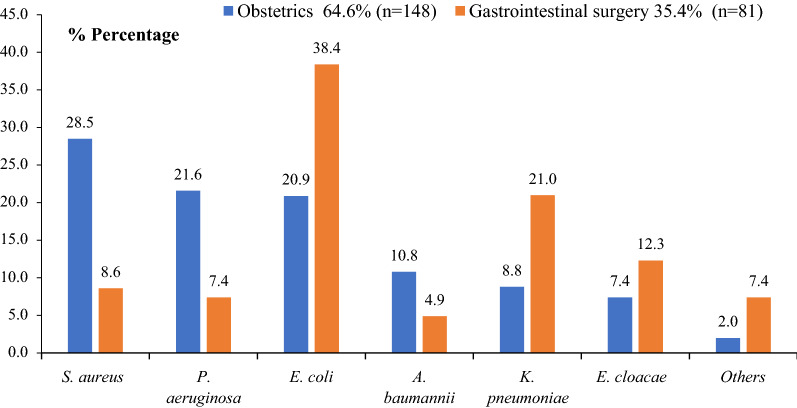
Proportion of bacterial species per ward: Obstetrics and Gastrointestinal

Among the 304 wound-swab cultures, 259 (85.2%) were positive for aerobic bacterial growth. Forty-five (n = 45,14.8%) yielded negative results, while 85 (27.9%) were excluded because they presented more than two germs and were considered to be polymicrobial. Of the 174 remaining samples, 55 (31.6%) yielded polymorph flora with two different bacteria, while 119 (68.4%) yielded a single isolate. Altogether, 229 isolates were identified. While Gram-positive microorganisms represented 21.4% (n = 49) of isolates, 78.6% (n = 180) were Gram-negative. Whereas *Staphylococcus aureus* (28.5%, n = 42), *Pseudomonas.aeruginosa* (21.6%, n = 32) and *Escherichia. coli* (20.9%, n = 31) were the most frequent in the obstetrics ward, *Escherichia. coli* (38.4%, n = 31), *Klebsiella pneumoniae* (21.0%, n = 17) and *Enterobacter cloacae* (12.3%, n = 10) were the most prevalent in gastrointestinal surgery (Figs. 1 and 2).

The genus was confirmed of all the bacteria we identified. No discrepancy appeared with *Staphylococcus aureus.* Two discrepancies each were noted with negative Gram bacteria: *Klebsiella pneumoniae* (API) versus *Klebsiella varicola* (MALDI-TOF), and *Pseudomonas aeruginosa* (API) versus *Pseudomonas mendocina (*MALDI-TOF).

### Drug resistance patterns of the isolates to different classes of antibiotics

#### Staphylococcus aureus

**Fig. 3:**
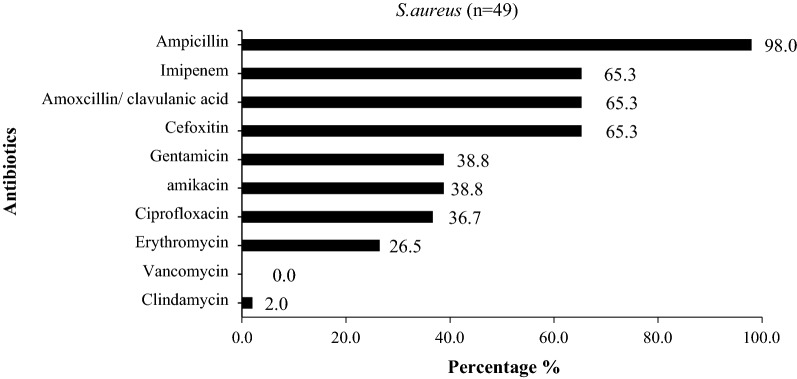
Antimicrobialresistance pattern among *S.aureus* isolated from pus specimens

All Gram-positive organisms were *S. aureus*. Almost all *S. aureus* isolates were resistant to penicillin (98%, n = 48/49), and 32 isolates (65.3%, n = 32/49) were resistant to methicillin (MRSA phenotype). Cefoxitin is considered to be a marker of the MRSA phenotype, and we observed the same rate of resistance in all beta-lactams classes. Resistance to cotrimoxazole was found in 10.2% of isolates, to gentamicin in 38.8%, and to ciprofloxacin in 36.7%. Ninety-eight percent of isolates were susceptible to clindamycin and no resistance to vancomycin was observed (Fig. 3).

#### Lactose fermenters bacteria

**Fig. 4:**
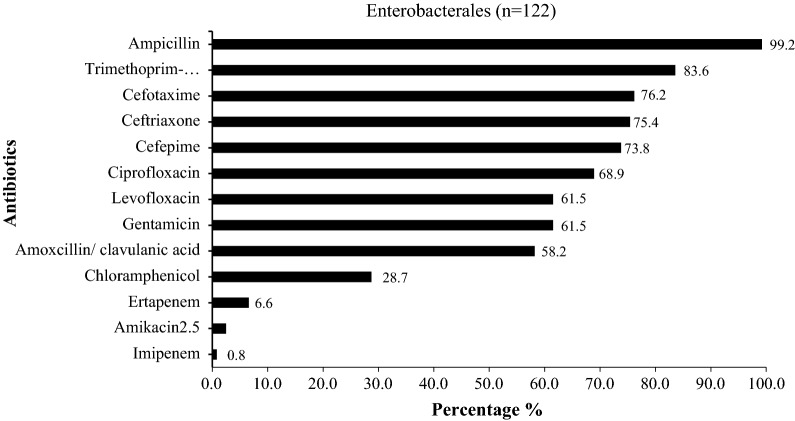
Antimicrobialresistance pattern among Enterobacterales isolated in pus

While all lactose fermenters bacteria (LFB) showed resistance to many of the antibiotics tested, amikacin and imipenem remained the most active (95.9% and 99.2%, respectively). However, there was also considerable resistance to other aminoglycosides (61.5% of LFB were resistant to gentamicin). Almost all LFB (99.2%) showed resistance to ampicillin. Three-quarters (75.4%) were resistant to ceftriaxone, 76.2% to cefotaxime and 73.8% to cefepime. Resistance to quinolones reached 68.9% for ciprofloxacin. There was also a high level of resistance to cotrimoxazole (83.6%). We noted 6.6% of LFB that were resistant to at least ertapenem (Fig. 4).

#### Lactose non-fermenters bacteria

**Fig. 5:**
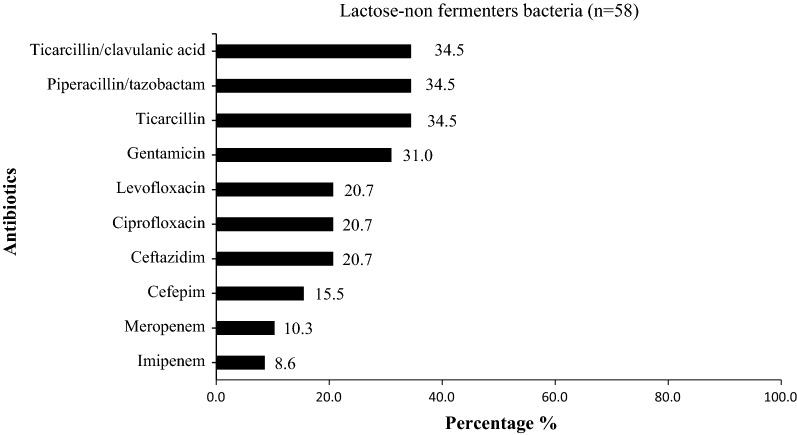
Antimicrobial resistance pattern among lactose non-fermenters bacteria

The lactose non-fermenters bacteria (LNFB) (*Acinetobacter baumannii* n = 20 and *P. aeruginosa* n = 38*)* showed low resistance to ciprofloxacin (20.7%), ceftazidime (20.7%), gentamicin (31.0%) and piperacillin/tazobactam (34.5%). Unfortunately, 8.6% of LNFB were resistant to imipenem and 10.3% of LNFB were resistant meropenem (Fig. 5).

### *Prevalence of ESBL and carbapenem-resistant isolates of gram negative rods*

Forty-three of the 62 isolates of *E.coli* (69.4%) were ESBL producers, as were 25 of the 30 (83.3%) *K. pneumoniae* isolates, and 16 of the 21 *E. cloacae* (76.2%) isolates. Five isolates (8%) of *E. coli* and three *E. cloacae* (14%) were resistant to at least one of the carbapenems tested (imipenem, meropenem and ertapenem)*.* For all of the ESBL strains (84), the percentage of co-resistance was 85.7% (72/84) for quinolones, 65.5% (55/84) for aminoglycosides, and 67.8% (57/84) for trimethoprim-sulfamethoxazole. Three isolates of *P.aeruginosa* (8%) and one of *A. baumannii* (5%) showed resistance to carbapenems. With the RESIST -3 O.K.N.ICT test, we detected New Delhi-Metallo-beta-lactamase (NDM) in *A.baumannii* and VIM in *P.aeruginosa.* According to the PCR, the ESBL isolates harbored mainly Cefotaximases-Munich (CTX-M) enzymes. The molecular description of these genes will be reported soon.

### Multidrug resistance pattern of bacterial isolates

As well as majorities of *E.coli* (93.5%) and *E. cloacae* (95.2%), 69.4% of *S. aureus* strains were resistant to more than five antimicrobial classes. All *P. aeruginosa* and *A. baumannii* strains were also resistant to more than five classes of antibiotic (Table 1).

**Table 1:** Multiple drug resistance patterns of the isolated bacteria

Isolates	Total (N)	R2 (%)	R3 (%)	R4 (%)	≥ R5 (%)
*E. coli*	62	0 (0)	1(1.6)	3 (4.8)	58 (93.5)
*S. aureus*	49	7 (14.3)	6 (12.2)	2 (9.1)	34 (69.4)
*P. aeruginosa*	38	0 (0)	0 (0)	0 (0)	38 (100)
*K. pneumoniae*	30	0 (0)	0 (0)	1(3.4)	29 (96.6)
*E. cloacae*	21	0 (0)	1(4.8)	0 (0)	20 (95.2)
*A baumannii*	20	0 (0)	0 (0)	0 (0)	20 (100)
Others	9	0 (0)	0 (0)	0 (0%)	9 (100)
Total	229	7 (3.1)	8 (3.5)	6 (2.6)	208 (90.8)

R2–R5 number of antibiotics class to which an isolate was resistant

#### Discussion

This study provides insight into the causative bacteria and sensitivity profiles of SSIs in six hospitals in Benin. Overall, 90.8% of aerobic bacteria were resistant to more than five antimicrobial classes—an MDRO rate that was higher than those described in Ethiopia and Uganda [23, 24].

Three main factors may have contributed to these high rates of MDRO. The first is likely to have been associated with the country’s overall lack of antimicrobial resistance surveillance and stewardship programs. There is enough evidence to indicate that, by improving the use of antibiotics, such programs help both to understand the pattern of resistance and to prevent the development of antibiotic resistance [6]. The second reason might be associated with the lack of comprehensive national policies on antibiotics use. Instead, it is common practice in Benin to buy antibiotics—including large-spectrum ones—without prescription from private drug vendors and pharmacies. The third reason may be due to the lack of diagnosic laboratory services before the administration of antibiotics by clinicians who do not have an antibiogram or evidence of the causative agents.

The most common isolates in obstetrics were *Staphylococcus aureus* and *Pseudomonas aeruginosa*. This finding is in line with other studies that described S. *aureus* to be associated with SSIs in obstetric wards [25–27]; *S. aureus* is considered to be a commensal organism of the skin and can easily contaminate a wound [28]. The high frequency of *P. aeruginosa* could be explained by the fact that this bacteria is intrinsically resistant to ceftriaxone, which is currently the drug most commonly used for prophylaxis in Benin.

According to the AST results, almost all *S. aureus* isolates (98%) were resistant to penicillin. While a similarly high resistance of *S. aureus* to penicillin was also reported in Uganda and Nepal [23, 26], resistance to ampicillin was observed in only 4% of isolates in India [29]. Such variations in the susceptibility pattern may be attributed to differences in the rational use of antibiotics. In Benin, ampicillin has also been widely used as a prophylaxis after ceftriaxone.

Two-thirds of our isolates (65.3%) were resistant to cefoxitin and were reported as MRSA species. Cefoxitin has been reported as a surrogate marker for the detection of methicillin resistance. The cefoxitin zone diameter remains an interpretive criterion for the prediction of mecA-mediated resistance [30]. Upreti and Shrestha in Nepal found the same rate of MRSA [31, 32]. In a retrospective single center study conducted in 2016 by Mercy Ship during surgical outreaches in six sub-Saharan African countries (Benin, Togo, Liberia, Madagascar, Congo and Sierra-leone), Lai PS et al. found the highest rates of MRSA in Benin (34.6%) and Congo (31.9%), and the lowest rate in Togo (14.3%) and Madagascar (14.5%) [33]. The difference in the rates of isolation of MRSA between studies may have been due not only to differences in the levels of inappropriate use of antibiotics, but also to the effectiveness of hygiene programs. In a previous study [34], we found hand-hygiene compliance among Benin healthcare providers to be only 33.3%. The treatment of infections caused by MRSA may also require the use of reserve drugs such as glycopeptids or lincosamides. However, the fact that we observed no resistance to vancomycin in our study can be explained by the fact that this antibiotic was not available in Benin.

*E. coli, K. pneumoniae* and *E. cloacae* were the commonest isolates in gastrointestinal surgery in our study. The predominance of *E. coli* has been reported in some other recent studies [35–37]. In Morocco and Uganda, the authors showed *K. pneumoniae* to be the predominant Gram-negative bacteria [23, 36]. This predominance could be attributed to their diverse habitats (which includes inanimate surfaces in hospitals), their multidrug resistance pattern, and possible contamination from the intestinal tract during surgery.

The bacteria most frequently involved in SSIs change from time to time and also vary with hospital settings. Our finding that GNB showed high resistance to ceftriaxone, ceftazidime, cefepime, and cotrimoxazole is in agreement with various studies worldwide [38–41]. The high rate of bacterial resistance to ceftriaxone is likely due to frequent use of this antibiotic in and outside hospitals. Our finding that almost all *P. aeruginosa* and *A. baumannii* were sensitive to amikacin and had relatively moderate resistance to cefepime (15.5%), ceftazidime (20.7%), and ciprofloxacin (20.7%) are similar to those in studies in Nepal and India [31, 32], which observed moderate resistance to ciprofloxacin (6.2% to 24%). High sensitivity to imipenem and amikacin may be due to the limited exposure of these drugs to the prescription antibiotics that are relatively more expensive and not constently available in Benin. For instance, amikacin is available for one case out of two; clinicians are forced to buy it from neighboring countries such as Nigeria and Togo.

The emergence of ESBL-producing Gram-negative rods has attracted increasing concern in the developing world [12]. The majority of LFB isolates in our study were ESBL producers. Upreti et al. reported that 25% of *E. coli* and 40% of *K. pneumoniae* are ESBL producers [32]. In 2016, Benin was found to have the highest rate of third-generation cephalosporin-resistant LFB of six sub-Saharan African countries [33]. Almost all ESBL producers showed co-resistance to other class of antibiotics, such as aminoglycosides (65.5%), quinolones (85.7%), and trimethoprim-sulfamethoxazole (67.8%). This high co-resistance may be due to the occurrence of gene-encoding resistance to aminoglycosides, trimethoprim-sulfamethoxazole and quinolones on the same plasmid that encode ESBL production [42].

Although patients in the present study received prophylactic antimicrobials such as ceftriaxone and ampicillin prior to surgery, the antibiogram results showed that the isolated organisms were resistant to these antimicrobial agents. Ceftriaxone is also known to favor the emergence of ESBL. This high antibiotic resistance implies that if immediate action is not taken, recommended antibiotics such as cefazolin may be rendered useless.

In our study, 10.3% of *P.aeruginosa* and *A. baumannii* showed resistance to meropenem and constituted pan-drug resistance bacteria according to the Magiorakos classification [19]. This resistance was due to mechanisms that are often expressed in hospital-acquired strains of *Acinetobacter* and *Pseudomonas*, such as beta-lactamases, alterations in cell-wall channel (porins), and efflux pumps. Due to the unavailability of an effective last therapeutic option such as ceftazidime-avibactam, the increasing rate of carbapenemase-producing organisms in this study is of great concern [43].

To the best of our knowledge, our report is the first from Benin on the rate of MDRO. Like other LMICs, Benin does not have a strongly regulated antibiotic-prescription system, which makes it particularly easy to misuse antibiotics. Our findings thus constitute an urgent call for monitoring and optimizing antimicrobial use there. Our first recommendation is for a multidisciplinary approach to the management of SSIs that involves clinicians, pharmacists, microbiologists and infection-control specialists. Second, strengthening laboratory services at the local and national levels would ensure effective surveillance of antimicrobial resistance. Finally, to minimize the spread of MDRO, we recommend strict adherence to good infection-prevention control practices, particularly hand hygiene and the disinfection of inanimate surfaces.

This study is part of the Multidisciplinary Strategy for Prevention and Infection Control in Benin (MUSTPIC). One challenge facing this strategy is the question of how, on the basis of our findings, national guidelines may be formulated for the correct application of SAP in our hospitals.

### Limitations of this study

While the strength of this study lies in its prospective nature, a limitation should also be noted. As our study did not isolate strict anaerobes, the number of bacterial isolates that were reported negative may have been underestimated. Relevant additional information would be produced by further studies. Molecular characterization of MDRO would have generated more useful epidemiological results.

#### Conclusion

This study helps quantify the extent of drug-resistant bacteria in surgical site infections in Benin. As two-thirds of isolates were producers of ESBL and MRSA, this prevalence particularly concerns GNB and *S. aureus*. None of the isolates showed a wild-type susceptible phenotype. With regard to reducing the spread of multidrug-resistant bacteria in Benin, these findings represent an urgent call for the judicious use of antibiotics, for strict adherence to good hand-hygiene practices, and for the provision of antibiotics with high activity. Even though research on AMR in Benin is still at a very early stage, it is essential to establish surveillance programs that reduce the burden of surgical site infections.

## Data Availability

The datasets used and/or analyzed during the current study are avalaible from the corresponding author.

## Abbreviations

AMR: Antimicrobial resistance
AST: Antimicrobial susceptibility test
ATCC: American type culture collection
ESBL: Extended-spectrum beta-lactamase
MDRO: Multi-drug resistance organisms
MRSA: Methicillin resistant *Staphylococcus aureus*
WHO: World health organization
LFB: Lactose fermenters bacteria
LNFB: Lactose non-fermenters bacteria
HAIs: Healthcare associated infections
SSI: Surgical site infection
SOPs: Standard operative procedures
SPSS: Statistical package for social sciences
LMIC: Low middle-income country
